# Biofilm delays wound healing: A review of the evidence

**DOI:** 10.4103/2321-3868.113329

**Published:** 2015-06-26

**Authors:** Daniel G. Metcalf, Philip G. Bowler

**Affiliations:** 1 Infection Prevention, ConvaTec Ltd., Global Development Centre, First Avenue, Deeside Industrial Park, Flintshire, CH5 2NU UK; 2 ConvaTec Global Development Centre, First Avenue, Deeside Industrial Park, Flintshire, CH5 2NU UK

**Keywords:** Biofilm, delayed healing, evidence, wound

## Abstract

Biofilm is the predominant mode of life for bacteria and today it is implicated in numerous human diseases. A growing body of scientific and clinical evidence now exists regarding the presence of biofilm in wounds. This review summarizes the clinical experiences and *in vivo* evidence that implicate biofilm in delayed wound healing. The various mechanisms by which biofilm may impede healing are highlighted, including impaired epithelialization and granulation tissue formation, and reduced susceptibilities to antimicrobial agents and host defenses. Strategies to manage biofilm and encourage progression to wound healing are discussed; these include debridement and appropriate antimicrobial therapies which may be improved upon in the future with the emergence of anti-biofilm technologies.

## Biofilm natural history and medical significance

Bacteria are ubiquitous in nature, and while the majority are harmless and important in life and for human health, some are capable of causing disease. Whether they develop within natural or pathogenic ecosystems, bacteria have a preference for existence as a surface-attached community, rather than as a planktonic (free-floating) mode of life. It has been suggested that bacteria evolved as surface-attached organisms, and that a planktonic phenotype subsequently evolved as a dispersal and seeding mechanism.[1]

The observation of bacterial aggregation and attachment to the surface of teeth was first made by van Leewenhoek in the late 1600s, but it was not until the 1970s, when researchers had access to more advanced microscopy and traditional microbiology techniques, that the true significance of surface-attached bacteria in ecosystems as diverse as the bovine stomach and alpine streams was realized. Investigation of granite rocks in a Canadian alpine stream revealed that the bacterial population embedded within a slippery slime layer on rock surfaces outnumbered planktonic bacteria in the steam water by a factor of 1,000-10,000;[2,3] this emphasizes the preference of bacteria to attach to surfaces. It was during this period that the term ‘biofilm’ was first used to describe surface-adherent bacteria encased within, and protected by a self-produced glycocalyx (today more commonly referred to as extracellular polymeric substance, or EPS).[3] Biofilm research since the 1970s has been extensive, with substantial evidence indicating that bacteria exist predominantly as a biofilm phenotype in medical, natural and industrial ecosystems.[3] The impact of biofilm in waste water filtration, biofouling of industrial materials, metal corrosion, and human chronic bacterial infections has been widely documented.

**Table:** 

Access this article online	
**Quick Response Code**:	**Website**: www.burnstrauma.com
	**DOI**: 10.4103/2321-3868.113329

Today, biofilm is implicated in numerous bacterial infections including those associated with the urinary tract, ear, sinuses, indwelling catheters, cystic fibrosis, periodontal disease and chronic wounds. Research undertaken during the 1970s, predominantly pioneered by J. William Costerton (the father of biofilmology), showed that ‘healthy’ biofilm on normal tissue surfaces provided protection against pathogenic colonization,[4] and that pathogenic biofilm predominated in infected tissues associated with chronic endocarditis and indwelling devices.[5] Additionally, it became evident that infections associated with bacterial biofilm persisted despite aggressive antimicrobial chemotherapy[6] Biofilm tolerance to antimicrobial agents[7] and host defense mechanisms[8,9] is now well documented, and this highlights the importance of effective biofilm management in chronic infections.

There is no doubt that biofilm exists in some wounds, and in the last decade a growing body of supportive evidence has emerged.[10,13] Table 1 summarizes some key scientific evidence for the presence of biofilm in wounds.[10,11,14–19] It is likely that at least half of all chronic wounds contain biofilm,[10,11] the implications of which are considerable. If a majority of non-healing wounds contain biofilm, and it has a role in delayed healing, then biofilm could be contributing many billions of dollars to the global cost of chronic wounds.[20,21] Recent evidence from animal models has demonstrated that biofilm creates a low-grade and persistent inflammatory response, and impairs both epithelialization and granulation tissue formation.[22] Additionally, due to clinical observations of suspected biofilm, specific biofilm-based wound management is now being practised.[12] This review will focus on the most recent clinical experiences and *in vivo* evidence from relevant animal models to summarize the latest knowledge of the effects of biofilm on wound healing.

**Table 1:** Key scientific evidence for the presence of biofilm in human wounds

Wound type	No.	Methods	Observations	Reference
Chronic wounds	50	Light microscopy, scanning electron microscopy (SEM)	30 (60%) chronic wounds observed to contain biofilm	James *et al.* (2008)[10]
Acute wounds	16	Light microscopy, SEM	1 (6%) acute wound contained biofilm	James *et al.* (2008)[10]
Chronic wounds	22	Confocal microscopy	13 (59%) chronic wounds contained biofilm	Kirketerp-Møller *et al.* (2008)[11
Chronic wounds	2	Fluorescence microscopy	Both samples contained biofilm	Bjarnsholt *et al.* (2008)[14]
Chronic wounds	10	Fluorescence microscopy, confocal microscopy	*Pseudomonas aeruginosa* biofilm seen deeper in wound bed than *Staphylococcus aureus*	Fazli *et al.* (2009)[15]
Chronic wounds	10	Fluorescence microscopy, confocal microscopy	*P. aeruginosa* biofilm elicited greater inflammation than *S. aureus*	Fazli *et al.* (2011)[16]
Mixed etiologies	15	Fluorescence microscopy	7 (47%) wounds contained biofilm	Han *et al.* (2011)[17]
Diabetic foot ulcers	2	Confocal microscopy	Both samples contained biofilm	Neut *et al.* (2011)[18]
Full-thickness burns	11	Light microscopy, transmission electron microscopy, SEM	Ulcerated areas and escharotomy sites contained biofilm; non-ulcerated areas did not	Kennedy *et al.* (2010)[19]

## Biofilm and wound healing

### Clinical evidence

**Figure 1:**
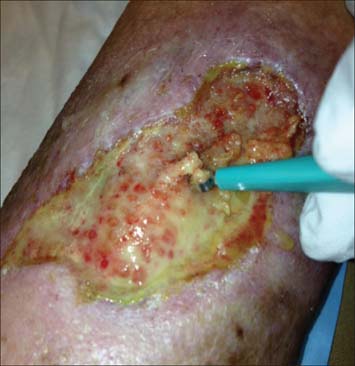
An infected traumatic leg ulcer in a diabetic patient with moderate peripheral arterial disease. Curettage was used to remove the pale yellow, slimy biofilm from the wound. Small buds of granulation tissue can be seen beneath the biofilm.

**Figure 2:**
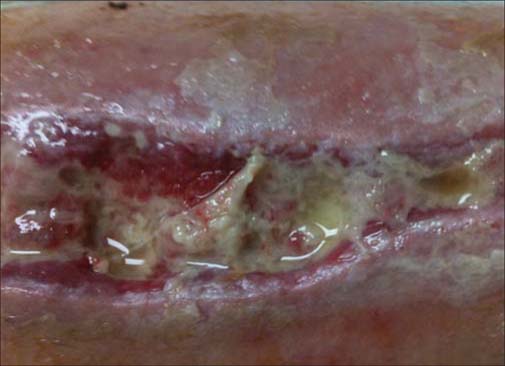
A dehisced surgical incision in a relatively ischemic patient. The opaque film on the wound bed (centre) re-formed daily and could be lifted off to reveal intact granular buds. Ultrasonic debridement was ineffective at disrupting or removing this thick, mature biofilm. Slough is also evident on the intact skin around the wound (top centre, top right, bottom right).

In a series of case studies, Hurlow (2009 and 2012) described management of wound biofilm using carefully selected combinations of debridement, antimicrobials and dressing technologies [Table 2].[13,23] Figure 1 shows an example of an infected traumatic wound undergoing curettage to debride biofilm that appeared to be impeding granulation tissue formation. Careful management of infection, exudate and underlying pathophysiologies were complemented by this approach.[23] Hurlow’s work also highlighted key differences between the origins, composition and behavior of slough and biofilm. Slough is dead or devitalized proteinaceous host tissue, but contiguous with underlying viable tissue.[13] On the other hand, biofilm is viable, bacteria-derived tissue, comprised of bacteria in a matrix of EPS which is thought to be primarily polysaccharide-based.[28] This distinction is exemplified in Figure 2 which shows a dehisced surgical wound with suggestive biofilm in addition to peripheral slough. The claim that biofilm is sometimes visible in wounds with the naked eye[23] has initiated lively debate amongst the global wound care community.[29] A shiny, translucent, slimy layer in the wound bed had already been used as a clinical sign of biofilm, especially if it returned quickly after sharp debridement.[30] It has recently been argued that there is no conclusive *in vivo* proof that biofilm exists in wounds *perse*^+^; however, the authors highlight the need for biofilm detection technology and support biofilm-based wound care (BBWC).[31]

**Table 2:** Clinical evidence that biofilm delays wound healing

Wound type	No.	Clinical observations	Biofilm management	Reference
Non-healing surgical ulcer	1	Cloudy, shiny, thin film of slime, after lavage, enzymatic ointment and a silver alginate dressing	Curettage gently scraped away film; managed underlying pathophysiology	Hurlow & Bowler (2009)[13]
Venous leg ulcer (VLU)	1	Thick, visible film, after lavage, collagenase debridement and a silver alginate dressing	Continual debridement plus negative pressure wound therapy and split-thickness graft	Hurlow & Bowler (2009)[13]
VLU	1	Persistent, cloudy, translucent film, after a silver alginate dressing	Sodium hypochlorite wound cleanser between dressing changes	Hurlow & Bowler (2009)[13]
Diabetic with cellulitis	2	Visible, opaque, pale yellow films	Antibiotics, debridement and a silver carboxymethyl cellulose dressing	Hurlow & Bowler (2012)[23]
Highly exuding	3	Thick, green-tinted or translucent film, after inappropriate dressings (polyurethane, hydrogel or foam dressings)	Two wounds healed using antibiotics, debridement and a silver carboxymethyl cellulose dressing	Hurlow & Bowler (2012)[23]
Peripheral arterial disease (PAD)	3	Cloudy and translucent film / opaque film / red/green film	Sharp debridement and a silver carboxylmethyl cellulose dressing	Hurlow & Bowler (2012)[23]
Critically ischemic lower limb wounds	190	77% (146) wounds healed	Combinations of sharp and ultrasonic debridement, lactoferrin/xylitol, cadexomer iodine and silver dressings	Wolcott & Rhoads (2008)[12]
Dehisced	4	Healing	Sharp debridement	Wolcott *et al.* (2010)[24]
Lower limb traumatic in a PAD patient	1	Wound had become chronic with suspected biofilm	Healed over 6 months using BBWC	Wolcott *et al.* (2010)[25]
Traumatic chemical burn in a diabetic	1	Infection developed and patient was declared an amputation case	Healed in 12 weeks using debridement, systemic and topical antibiotics and silver dressings	Wolcott & Dowd (2011)[26]
Mixed etiologies undergoing cell-based therapy	97	Entire graft material remained intact with biofilm suppression	Debridement and personalised topical gels containing anti-biofilm agents and antibiotics	Wolcott & Cox (2013)[27]

In recent years, Wolcott and colleagues have embarked on a series of clinical investigations which have advanced our understanding of the characterization, behavior and impact of biofilm in chronic and acute wounds [Table 2].[12,24–27]

**Figure 3:**
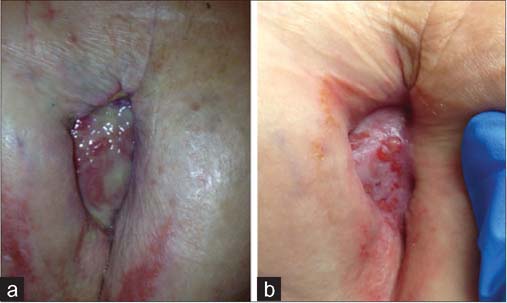
A dehisced mastectomy incision wound in a patient who had undergone chemotherapy. (a) Debridement in conjunction with a silver carboxymethyl cellulose dressing was used to transform a biofilm colonized wound. (b) Debridement in conjunction with a silver carboxymethyl cellulose dressing was used to transform a healing wound.

BBWC is an algorithm, including debridement, antimicrobial dressings, anti-biofilm agents and antibiotics, used to most efficiently suppress wound biofilm and encourage healing. In a retrospective clinical study, the improved level of healing achieved using BBWC was found to be statistically significant.[12] The value of regular debridement was demonstrated in a series of scientific, animal and clinical analyses, which showed that debridement opens up a therapeutic window where bacteria are more susceptible to antimicrobial agents,[32] so is key to maintaining a healthy wound bed.[33] A recent retrospective study examined a group of 97 patients with various wounds that received BBWC in conjunction with cell-based therapy. Compared to previous studies which did not use BBWC, these bespoke protocols of care were shown to result in a statistically significant improvement in wound healing following cell-based therapy[27] Figure 3 shows an example of how debridement in conjunction with a silver carboxymethyl cellulose dressing was used to successfully transform a biofilm-colonized dehisced incision [Figure 3a] to a healing wound [Figure 3b].

These clinical experiences, case studies and retrospective studies are of great value as we begin to understand the effect of biofilm on wound healing. However, in the absence of a non-invasive, point-of-care biofilm detection method, it is challenging to use human patients to conduct randomized or prospective trials on the subject. It is also unethical to consider deliberate establishment of biofilm in human patients. Therefore, the use of animal models is justifiable in investigating this significant human health problem. Moreover, certain animal wound models are relevant to human wound healing in terms of host response such as the production of macrophages, neutrophils, inflammatory enzymes and keratinocytes. Porcine, murine and rabbit ear wound models are all recognized for their value in better understanding human wound pathophysiology and healing.

### Animal evidence

Studies using the porcine acute wound model suggested that biofilm physically impairs the immune responses associated with healing [Table 3].[34,35] The authors described how continuous antimicrobial treatment was needed to control biofilm-colonized wounds, and that debridement was a critical step to reduce bioburden before therapeutics are applied,[49] as has been described clinically[32,33] A porcine model has demonstrated that encouraging the establishment and resilience of wound biofilm exacerbates delayed healing [Table 3].[36] Most recently, the interaction between *S. aureus* and *P. aeruginosa* in biofilms has been demonstrated in the porcine model where healing was significantly delayed [Table 3].[37] Murine models have been used to demonstrate the importance of *P. aeruginosa biofilm*-forming virulence factors,[38] and that EPS protects bacteria from antibiotics and host defenses [Table 3].[39] The diabetic mouse chronic wound model has shown that *P. aeruginosa* biofilm significantly delayed healing by two to four weeks without impairing the general health of the subject [Table 3].[40,41] Treating diabetic mice with insulin did not prevent delayed healing, actually promoting *P. aeruginosa* biofilm [Table 3],[42] which has also been shown to be resistant to undiluted sodium hypochlorite.[32] Polymicrobial biofilm comprised of four species delayed murine wound closure significantly more than single-species biofilm over 12 days [Table 3].[43]

**Table 3:** Animal evidence that biofilm delays wound healing from porcine, murine and rabbit ear wound models

Model	Biofilm species	Observations	Reference
Porcine acute wound	*S. aureus*	Challenge with antimicrobial agents confirmed the recalcitrance of biofilm bacteria	Serralta *et al.* (2001)[34]
Porcine acute wound	*S. aureus*	Indirect evidence of delayed healing, with polymorphonucleocytes observed on the surface of, but not within, biofilm	Davis *et al.* (2008)[35]
Porcine acute wound	Methicillin-resistant *S. aureus* (MRSA)	Greater healing delays were observed due to biofilm formed by passaged MRSA strains than by parent strains; passaged strain was observed to form more biofilm than parent strain	Roche *et al.* (2012)[36]
Porcine partial-thickness wound	MRSA, *P . aeruginosa*	Interactions between MRSA and *P. aeruginosa* were observed, delaying healing due to suppression of epithelialization and expression of virulence factors	Pastar *et al.* (2013)[37]
Murine burn	*P. aeruginosa*	A biofilm-forming factor established *in vitro* was a key virulence factor *in vivo*	Rashid *et al.* (2000)[38]
Murine burn	*P. aeruginosa*	Microscopic biofilm that was not readily removed by rinsing with saline	Schaber *et al.* (2007)[39]
Murine diabetic chronic wound	*P. aeruginosa*	(*In vitro* then inoculated) biofilm significantly delayed healing compared to controls; health parameters in biofilm-colonized mice were normal	Zhao *et al.* (2010)[40]
Murine diabetic chronic wound	*P. aeruginosa*	(*In vitro* then inoculated) biofilm-colonized wounds had high levels of inflammatory cells; 8 weeks for all biofilm-colonized wounds to heal, compared to 4 weeks for controls	Zhao *et al.* (2012)[41]
Murine diabetic chronic wound	*P. aeruginosa*	Biofilm significantly delayed wound healing, even in diabetic mice treated with insulin	Watters *et al.* (2012)[42]
Murine chronically infected surgical wound	*P. aeruginosa*	Biofilm was highly resistant to antibiotics and undiluted sodium hypochlorite once established over several days	Wolcott *et al.* (2010)[32]
Murine chronically infected surgical wound	*S. aureus*, *P. aeruginosa*, *Enterococcus faecalis*, *Finegoldia magna*	(*In vitro* then inoculated) polymicrobial biofilm was maintained for 12 days, and delayed healing more than *P. aeruginosa* biofilm, as measured by wound closure	Dalton *et al.* (2011)[43]
Murine splinted wound	*S. aureus* or *Staphylococcus epidermidis*	Biofilms significantly delayed epithelialization; inhibition of biofilm restored normal wound healing	Schierle *et al.* (2009)[44]
Rabbit ear wound	*S. aureus*	Biofilm and active infection significantly delayed epithelialization and granulation tissue formation; biofilm-colonized wounds expressed significantly lower levels of inflammatory cytokines than infected wounds	Gurjala *et al.* (2011)[22]
Rabbit ear wound	*P. aeruginosa*	Biofilm significantly delayed epithelialization and granulation tissue formation; debridement, lavage and silver sulphadiazine in combination were more effective at restoring healing than individua treatments	Seth *et al.* (2012a)[45]
Rabbit ischemic ear wound	*Klebsiella pneumoniae*	Biofilm formed readily in ischemic wounds but not in non-ischemic wounds where neutrophils and macrophages were seen	Seth *et al.* (2012b)[46]
Rabbit ischemic ear wound	*K. pneumoniae*, *S. aureus*, *P. aeruginosa*	*K. pneumoniae* biofilm was least virulent, *P. aeruginosa* biofilm most virulent, measured by healing inhibition and inflammatory responses; EPS-deficient *P. aeruginosa* did not delay healing	Seth *et al.* (2012c)[47]
Rabbit ear wound	*S. aureus*, *P. aeruginosa*	Two-species biofilm elicited significantly elevated inflammatory response and impaired epithelialization and granulation tissue formation compared to single-species biofilm	Seth *et al.* (2012d)[48]

The Northwestern University group in Chicago has conducted a series of *in vivo* studies in recent years, expanding our understanding of the effect of biofilm on wound healing [Table 3]. Visible biofilm was observed to significantly delay closure of the epithelial gap in a murine splinted wound model [Table 3],[44] before an established, reproducible and Food & Drug Administration (FDA)-recognized rabbit dermal ulcer model of wound healing was adopted.[50] This model utilizes full-thickness punch wounds through to the cartilage of rabbit ears, closely representing the dermal damage observed in human chronic wounds. Biofilms of *S. aureus*[22] and *P. aeruginosa*[45] significantly delayed healing in terms of epithelialization and granulation tissue formation, with biofilm-colonized wounds expressing significantly lower levels of inflammatory markers than clinically infected wounds [Table 3]. Combinations of treatments in an anti-biofilm protocol of care were also shown to be more effective at encouraging wound healing than individual treatments [Table 3].[45] Biofilms of the opportunistic pathogen, *Klebsiella pneumoniae*, associated with burn and war wounds, were shown to impair healing of ischemic rabbit ear wounds,[46] but to a lesser extent than *P. aeruginosa* or *S. aureus* [Table 3].[47] A further study demonstrated synergy between different species in biofilm, further delaying wound healing compared to single-species biofilm [Table 3],[48] as has been demonstrated in murine models.[43]

Despite the limitations of the rabbit ear wound healing model (e.g. an acute wound modeling a human chronic wound), its advantages over other animal models are clear and have recently been the subject of a review[51] As a highly controlled *in vivo* wound biofilm model, the rabbit ear model is also useful for assessing new anti-biofilm or antimicrobial technologies.[52,53]

## Management of wound biofilm

Based on the clinical and *in vivo* experiences summarized above, it is possible to devise effective anti-biofilm strategies, similar to BBWC, to encourage wound healing in everyday clinical practice. Indeed, the Tissue-Inflammation/Infection-Moisture-Edge (TIME) concept has recently been updated to include biofilm management as a key consideration in wound bed preparation.[54]

### Debridement

Physical debridement of foreign material is clearly the simplest, and currently the most effective method to remove these impediments to healing. Whilst clinicians have long appreciated that debridement of slough can encourage healing, the evidence suggests that this process also removes bacteria, in the form of contaminated or colonized slough, as well as biofilm. Debridement techniques range from specialist surgical and sharp debridement, gentler mechanical debridement with curettes, fabric pads, lavage or ultrasound, to autolytic debridement with moisture-retentive dressings.[55] There may also be a place for chemical debridement using rinse solutions or gels containing antiseptics such as sodium hypochlorite or hypochlorous acid.[56,57] Whichever method is utilized, the main clinical and *in vivo* observations are that biofilm re-forms rapidly-certainly daily, and likely within hours,[58] so regular debridement is key.[32,33] In addition, whereas slough may be contiguous with healthier underlying host tissue, biofilm may be more surface-associated so may respond well to gentler methods of debridement such as curettage, fabric pads or skin-safe chemical rinses. Moreover, although the aim of debridement is to remove devitalized tissue and ‘beat back’ biofilm to stop it re-forming,[32,33] it will only be effective if followed up with appropriate antimicrobials and wound management products.

### Topical antimicrobials

The abundance of currently-available antimicrobial agents (e.g. antibiotics, cleansers, gels, dressings) may be confusing to healthcare professionals. Antibiotics should be used responsibly and only when clinical infection is suspected or confirmed by clinical and microbiological assessment. Effective debridement of biofilm removes some of the protection bacteria are offered by EPS, forcing the remaining bacteria to revert to a more metabolically active form, so antibiotics (which are designed to kill planktonic bacteria), and topical antiseptics such as silver, iodine and polyhexamethylene biguanide (PHMB), are made more effective.[32,33] Perhaps as important as the antiseptic selected is the delivery vehicle used, which must interact optimally with the wound microenvironment. For example, in an exuding wound with suspected biofilm, a highly-absorbent antiseptic dressing should be used after effective debridement-the use of antiseptic gauze or mesh would be inappropriate, due to their poor exudate management capabilities. Prudent combinations of debridement, antimicrobials and wound management products is currently the best available protocol of care for wounds with suspected biofilm or infection.[12] However, the anti-biofilm efficacy of most currently available topical antimicrobial products is limited.

### Anti-biofilm agents

As we begin to appreciate the source (bacterial), composition (EPS or ‘slime’) and behavior (re-forms quickly) of wound biofilm, opportunities to improve on current wound care are presented. For example, in the future it may be possible to formulate wound care products, such as debridement pads, rinses or dressings, with agents that penetrate through biofilm EPS, thus exposing the bacteria and increasing their susceptibility to antimicrobials. Detergent-type agents could help to remove biofilm from the wound bed, or chemicals could be used that weaken the matrix to collapse biofilms which could then be mopped up by absorbent dressings. A number of such potential anti-biofilm agents have been proposed, such as xylitol, lactoferrin and ethylenediaminetetraacetic acid (EDTA),[12] but convincing clinical or *in vivo* evidence for their efficacy is lacking. A key challenge to science and industry is to better understand the composition of wound biofilm in terms of polysaccharide, protein, extracellular DNA and ions, in order to formulate anti-biofilm technologies. At least daily, we all use combinations of debridement, detergents and antimicrobials to manage oral plaque biofilm and maintain oral hygeine,[59] and this multi-modal approach is most likely how wound biofilm can be effectively managed to encourage wound healing.

### Biofilm detection

The current accepted gold standard for biofilm detection in wounds is by microscopic examination using expensive and specialized techniques such as confocal microscopy or electronic microscopy. Although in some instances, biofilm may be visible to the trained clinical eye,[13,23,30] the need for wound biofilm detection techniques for use at the point-of-care is clear.[31,54,60] Biofilm detection would enable more effective wound bed preparation techniques if the clinician could visualize if and where biofilm is present in the wound, perhaps by staining or tagging biofilm components to render them visible.[60] This would also enable the most appropriate and effective selection of antimicrobials and dressings, with associated cost savings, which are becoming increasingly important in global healthcare.

## Conclusion

By taking into account the growing body of scientific and clinical evidence regarding wound biofilm, this review has highlighted a multitude of mechanisms by which biofilm may be implicated in delayed healing. Biofilm is associated with impaired epithelialization and granulation tissue formation, and promotes a low-grade inflammatory response that interferes with wound healing. Polymicrobial biofilms, which invariably exist in chronic wounds, have been shown to delay healing to a greater extent than single-species biofilms. Taking these effects into account, wound biofilm likely evolves as a cryptic ecosystem that at some point is sufficiently established to interfere with wound healing, and if not managed effectively may progress to infection.

From a therapeutic perspective, multi-modal approaches to wound management, particularly involving frequent physical debridement and antimicrobial therapy have been shown to enhance healing to a greater extent than single therapies in both animal and human studies. Whilst frequent physical removal of wound biofilm and appropriate antibiotic and topical antimicrobial therapies are perhaps best practice today, there is clearly a need for new medical devices (including dressings) that are able to interfere with the complex biofilm communities that exist in non-healing wounds.
